# Congenital spinal tumor in a patient with encephalocele and hydrocephalus: a case report

**DOI:** 10.1186/1752-1947-5-9

**Published:** 2011-01-14

**Authors:** Farid Radmanesh, Farideh Nejat, Fatemeh Mahjoub, Mostafa El Khashab

**Affiliations:** 1 Department of Neurosurgery, Children's Hospital Medical Center, Tehran University of Medical Science, Tehran, Iran; 2 Department of Pathology, Imam Khomeini Hospital, Tehran University of Medical Sciences, Tehran, Iran; 3 Department of Neurosurgery, Hackensack University Medical Center, New Jersey, USA

## Abstract

**Introduction:**

Encephalocele is a rare congenital abnormality of the central nervous system, where brain tissue protrudes from a defect in the skull. Some anomalies are associated with encephalocele. However, the association of spinal teratoma and encephalocele has not been reported in the English literature.

**Case presentation:**

We report the case of an Iranian girl with a history of encephalocele surgery, who, at the age of four years, developed an intramedullary spinal teratoma, and discuss the pathogenesis of this association.

**Conclusion:**

To the best of our knowledge, this is the first report of an association between encephalocele and spinal teratoma.

## Introduction

Encephalocele refers to a group of rare congenital anomalies of the central nervous system (CNS), where brain tissue protrudes from a defect in the skull [1]. Its prevalence has been estimated to be 0.8 to four in every 10,000 live births [2].

Teratomas are tumors derived from all three germ layers [3]. In children, teratomas are more commonly found in the sacrococcygeal region than in the spinal cord [4], which occurs in one of 38,500 viable births. Intramedullary spinal teratomas are rare tumors [5]. In 41.7% of teratomas, a concomitant anomaly of the vertebral canal is found, most commonly a diastematomyelia, [4]. However to the best of our knowledge, there is nor repot of an association with encephalocele in the English literature.

We report a case of encephalocele and lumbar intramedullary teratoma and discuss the possible etiology.

## Case presentation

A four-year old Iranian girl was referred to the neurosurgical department with severe back pain and motor regression. She was the second child of nonconsanguineous parents, and was delivered by elective Cesarean section due to being repeat. She had a history of occipital encephalocele, which was treated surgically during the neonatal period and she later received a shunt to treat progressive hydrocephalus. She could sit at nine month of age and stand at two years, but was unable to walk. Six months before her referral, she had developed back pain, which was particularly severe at night, and after three months, she was unable to stand.

On physical examination, our patient was found to be generally normal, with good mental performance, and normal results from a neurological examination of the arms. She had a head circumference on the 75th percentile and a functional ventriculoperitoneal shunt. She could move her legs, but was unable to keep them up against gravity. Her deep tendon reflexes in the legs were exaggerated, and her sensory level was undetectable. She had urinary and fecal incontinence.

**Figure 1:**
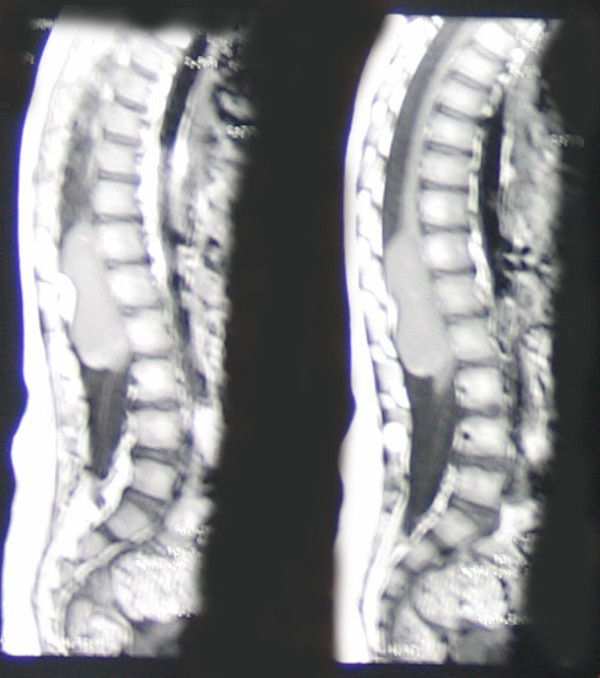
**Sagittal T1-weighted MRI scan showing an isointense tumor with fat signal on the dorsal surface**.

**Figure 2:**
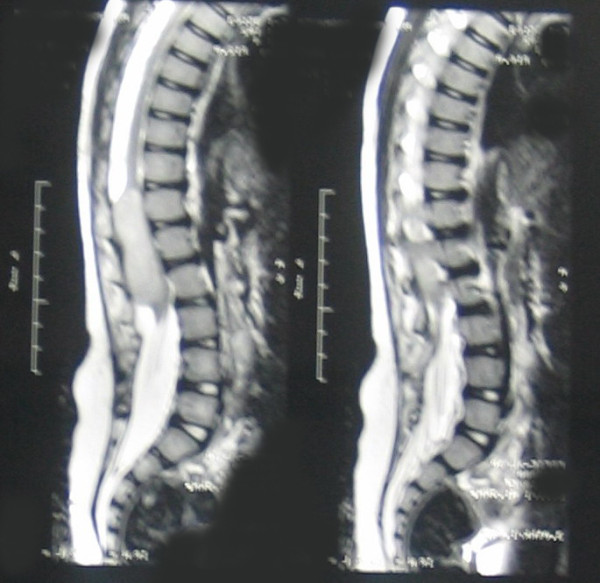
**Sagittal T2-weighted MRI scan showing an isointense mass in the thoracolumbar area**.

Spinal MRI revealed an intradural mass (Figure 1, Figure 2) extending from the T11 to T12 junction to the lower border of L2 vertebra. It was isointense on T1- and T2-weighted images, with a small piece of tissue on the dorsa of the mass, which was identified as lipoma.

The child underwent an osteoplastic laminotomy extending from T11 to L2. The dura matter was severely tense at the level where the laminotomy was opened. There was a white to creamy mass that was extramedullary at the distal level but intramedullary at the L1 and T12 levels. There was no real capsule around the mass, which contained small fine hairs and creamy fatty material. There was a small lipoma on the dorsal surface of the mass at the level of the L1 spine body.

**Figure 3:**
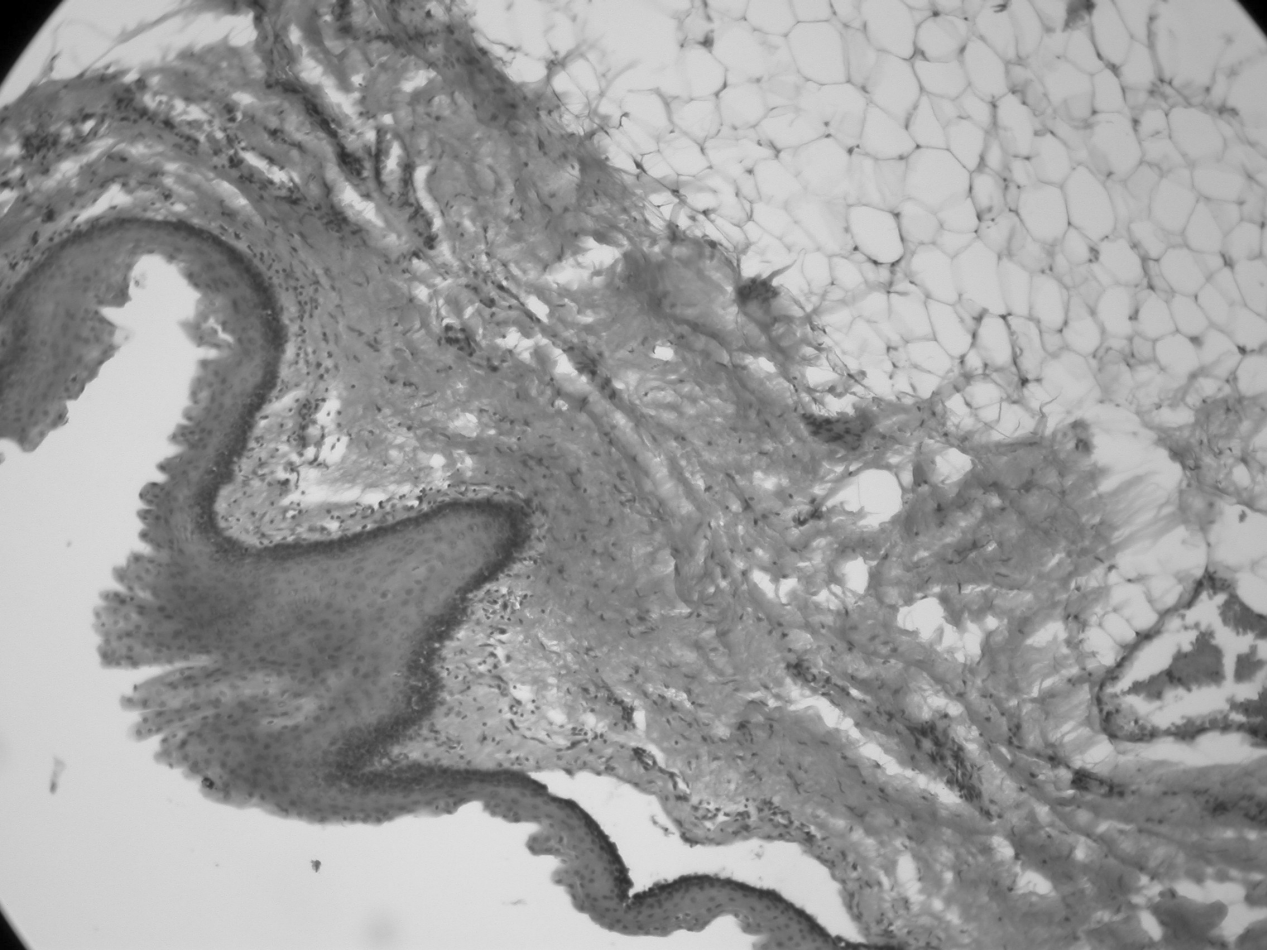
**Variety of tissues including skin, fat, connective and adipose tissue, and vascular structures (haematoxylin and eosin, original magnification × 40)**.

The lesion was excised completely. Histopathologic examination of the mass revealed a variety of tissues including skin, fat, connective and adipose tissue, and vascular structures (Figure 3). A pathological diagnosis of mature teratoma was made.

Our patient's post-operative period was unremarkable. One year after the operation she was able to stand by herself and to walk with the aid of a brace and walker. She was continent during the day but had nocturia.

## Discussion

Encephalocele is a cystic congenital malformation in which the cranial contents herniate through a defect in the cranium. Although encephalocele is typically classified as a neural tube defect, its underlying mechanism may differ from that of myelomeningocele, and probably occurs after neural tube closure [1]. Encephaloceles may present alone or in association with other congenital nervous system anomalies [1]. The presence of an intramedullary teratoma in association with encephalocele has not been reported previously.

Teratomas are tumors composed of derivatives of all three germ cell layers, and can be classified into mature and immature types based on the degree of differentiation [3]. The overall frequency of teratoma is one in 13,000 [3]. The origin of teratomas of the spinal cord is controversial. There are various theories on the pathogenesis of teratomas. The traditional view is that intraspinal teratomas arise from primordial germ cell misplaced from the primitive yolk sac, most commonly into midline structures [5]. In a more recent review of literature, Koen *et al*. suggested that a dysembryogenic process forms the basis of development of teratoma, especially those arising from spinal dysraphism. They proposed that the combination of mutated genes important for normal early neural development and cellular differentiation, and/or absent or deficient inductive signals, can lead to the formation of teratoma [6]. Moreover, the abnormal genetic and molecular pathways that result in the formation of encephalocele remain unclear. Although the possibility of two different pathogenesis cannot be excluded, it is more likely that the same genetic and molecular defects are responsible for this spectrum of findings. It is possible that these defects, which are present throughout the neuraxis, result in these two congenital anomalies, although they are theoretically formed during different stages of development. Because teratoma causes symptoms mainly through its mass effect as a result of progressive growth, there is a delay in symptoms becoming apparent.

This case emphasizes that, when dealing with a patient with a congenital anomaly who presents with new signs and symptoms or loss of developmental abilities that had already been acquired, it is essential to investigate if the new symptoms are due to causes other than the already existing anomaly, as in our patient. It is possible that another anomaly may be causing the symptoms, and the necessary investigations should be performed.

## Conclusion

In any patient with a congenital central nervous system anomaly who presents with new neurologic problems, the possibility of another anomaly, especially those that are believed to arise from the same pathogenic pathway, should be considered.

The exact pathogenic pathway of association between encephalocele and spinal teratomas remains to be elucidated. Although the possibility of two different pathogeneses could not be ruled out in our patient, it is more likely that the same genetic and molecular defects are responsible for this spectrum of findings. Further studies are needed to elucidate the probable genetic and molecular defects underlying these conditions.

## Competing interests

The authors declare that they have no competing interests.

## Consent

Written informed consent was obtained from the parents of the patient for publication of this case report and any accompanying images. A copy of the written consent is available for review by the Editor-in-Chief of this journal.

## Authors' contributions

FR and FN made major contributions in patient care, literature review and drafting of the manuscript. MEK made a substantial contribution to the literature review, correction and final approval of the manuscript. FM made the pathological exam and description. All authors read and approved the final manuscript.
